# Comfort for Suffering Feet

**Published:** 1876-03

**Authors:** 


### *COMFORT FOR SUFFERING FEET.*

If one member suffers, all the rest suffer, more or less, in partnership with it. The most suffering in the make-up of the human system is in that much-abused wonder of anatomy and construction—the foot. The feminine lungs, it is true, have been subjected to much torture from corsets; but even in this, womanhood is exempt from torture during its earliest years. The foot, whether masculine or feminine, adult or juvenile, has been squeezed, cramped, bent, and in divers ways maltreated from the cradle to the grave.

Under the old plan of shoeing the human foot, unthinking blunderers have done and are doing their most cruel deeds in trying to crowd the wonderfully made member into the shape of a wooden last of their own devising, and not in any reasonable respect like the shape of a natural foot. They seem to have considered the foot as a sort of wax-work or a graven image, or a something or other which should be in a state of continued repose.

The absurd foot-covering which they have and are making are fit only for exhibition in shop windows, and there they should be exhibited as curiosities of construction, and not as garments to be worn. Consequently there has been a continual antagonism between the foot and the leather case. The foot has been like a prisoner trying to break jail.

When Joel McComber, after years of thorough and patient study of the anatomy and mechanism of the human foot, first introduced his improvements in the way of his patent lasts, and shoes made on them to meet the requirements of the foot, some people laughed at him for an enthusiast, and others sneered at what they called his "hobby." But in the years which have now elapsed since that time, men of every walk in life and some whose walk was half-way between a painful limp and a miserable hobble, have tried his boots and found, both for their own feet and for those of their families, relief from long-accustomed torture. They have found that with the use of a decently-made and comfortable boot there is an improvement in general health and spirits, and a capacity for the accomplishment of more work and better work than could be done while the foot was being distorted and abused. They have found that Mr. McComber's plan of shoe-making is a necessity for children as well as for adults, and calculated to insure exemption from those distortions of which children have been the victims.

Mr McComber's business has now increased to such an extent that he has moved over to the fashionable side of Union Square, and taken the commodious and beautifnl store corner of Broadway and Fifteenth Street, the entrance being on Fifteenth Street. His business is here conducted by himself, having no partner and with ample room to accommodate his increasing hosts of patrons. He has made proof of his ability to do all that he claims. His word is accepted by the medical faculty as authority on feet and feet-coverings. His pamphlets on the feet of the people and their children ought to be read by everybody who has a child or a foot. They will be sent free to any address on application to him by letter or otherwise. And, having fully indorsed him before, we are now, after longer experience, prepared to indorse him more fully, and to advise all the men and women and children who come under our influence either to go barefoot or to wear the shoes of McComber.

